# fMRI at High Spatial Resolution: Implications for BOLD-Models

**DOI:** 10.3389/fncom.2016.00066

**Published:** 2016-06-28

**Authors:** Jozien Goense, Yvette Bohraus, Nikos K. Logothetis

**Affiliations:** ^1^Department of Psychology, Institute of Neuroscience and Psychology, University of GlasgowGlasgow, UK; ^2^Department of Physiology of Cognitive Processes, Max-Planck Institute for Biological CyberneticsTübingen, Germany; ^3^Divison of Imaging Science and Biomedical Engineering, University of ManchesterManchester, UK

**Keywords:** high-resolution fMRI, neurovascular coupling, cortical layers, BOLD mechanism, cerebral blood flow, cerebral blood volume

## Abstract

As high-resolution functional magnetic resonance imaging (fMRI) and fMRI of cortical layers become more widely used, the question how well high-resolution fMRI signals reflect the underlying neural processing, and how to interpret laminar fMRI data becomes more and more relevant. High-resolution fMRI has shown laminar differences in cerebral blood flow (CBF), volume (CBV), and neurovascular coupling. Features and processes that were previously lumped into a single voxel become spatially distinct at high resolution. These features can be vascular compartments such as veins, arteries, and capillaries, or cortical layers and columns, which can have differences in metabolism. Mesoscopic models of the blood oxygenation level dependent (BOLD) response therefore need to be expanded, for instance, to incorporate laminar differences in the coupling between neural activity, metabolism and the hemodynamic response. Here we discuss biological and methodological factors that affect the modeling and interpretation of high-resolution fMRI data. We also illustrate with examples from neuropharmacology and the negative BOLD response how combining BOLD with CBF- and CBV-based fMRI methods can provide additional information about neurovascular coupling, and can aid modeling and interpretation of high-resolution fMRI.

## High-resolution fMRI

High-resolution fMRI is gaining widespread interest because it allows more accurate spatial mapping of brain responses, for instance, finger- and digit representations in somatosensory cortex, orientation- and ocular dominance columns in primary visual cortex (V1), the fine-grained architecture of subcortical structures such as the lateral geniculate nucleus (LGN) or superior colliculus (SC), or the glomeruli and layers in the rat olfactory bulb (Cheng et al., 2001; Duong et al., 2001; Kida et al., 2002; Zhao et al., 2005; Chen et al., 2007; Yacoub et al., 2007, 2008; Zhang et al., 2010, 2015; Sanchez-Panchuelo et al., 2012; Wang et al., 2013; Siero et al., 2014; Poplawsky et al., 2015). fMRI of the cortical layers has been shown in rats, cats, and monkeys (Silva and Koretsky, 2002; Goense and Logothetis, 2006; Harel et al., 2006; Zhao et al., 2006; Zappe et al., 2008a; Goense et al., 2010; Yu et al., 2014) and with the advent of ultra high field human scanners (7T and higher), resolving cortical layers is becoming accessible in humans, evidenced by the increasing number of studies focused on high-resolution fMRI in humans (Koopmans et al., 2011; Huber et al., 2014, 2015; De Martino et al., 2015; Muckli et al., 2015). High-resolution fMRI of the cortical layers holds great promise as a tool for resolving cortical circuits *in vivo*, since many neural processes, such as feedforward vs. feedback, are segregated by layer (Felleman and Van Essen, 1991).

High-resolution fMRI presents challenges as well as opportunities. In humans, high-resolution fMRI usually refers to submillimeter fMRI, while in animals resolutions from 50 μm to a few 100 μm are considered high resolution (Logothetis et al., 2002; Silva and Koretsky, 2002; Harel et al., 2006; Zhao et al., 2006). Technical challenges are the limited signal-to-noise ratio (SNR), the need to simultaneously maintain temporal resolution and (whole brain) coverage, the increased sensitivity to motion and distortion, and for human scanners, the limitations on gradient strength and slew rate. High-resolution fMRI furthermore increases the sensitivity to inter-regional (Vigneau-Roy et al., 2014) and inter-individual anatomical differences. The increase in SNR and the increase in BOLD signal at high field allow higher fMRI resolution, and increasing the image resolution has been the driving force behind the development of ultra high field scanners for humans and animals. fMRI sensitivity is also limited by physiological noise, which determines to a large extent the temporal SNR (tSNR), although here there are also benefits of higher resolution, higher field and improved coil design (Triantafyllou et al., 2005; Goense et al., 2010).

Increasing the spatial resolution has implications for fMRI models of cortical processing. High-resolution fMRI starts to reveal the vascular and metabolic heterogeneity of the cortex, since the relevant features (pial and intracortical vessels, cortical layers) are of a similar size as the resolution (tens to hundreds of μm). To accurately predict high-resolution BOLD responses, some of these features need to be modeled explicitly (Heinzle et al., 2016; Markuerkiaga et al., 2016). A typical voxel size in human fMRI is 3 × 3 × 3 mm^3^, which spans multiple cortical columns and usually covers the entire cortical thickness with its associated vasculature; i.e., elements of the vascular system such as pial arteries and veins are lumped into the voxel, allowing for simpler models. In high-resolution fMRI these features become distinct, for instance, pial vessels can be visualized separately from cortical tissue, and at 100–150 μm resolution, penetrating arterioles and venules have been visualized in rats (Yu et al., 2012). Similarly, different responses in cortical columns and layers have been shown in cats, monkeys and humans (Cheng et al., 2001; Duong et al., 2001; Harel et al., 2006; Yacoub et al., 2007; Goense et al., 2012). The challenge however is that as these processes become distinct, such mesoscopic models need a larger number of compartments to model the BOLD response.

The question we aim to address here is: what additional compartments and terms should be included in mesoscopic fMRI models to explain or predict high-resolution fMRI data? Additional terms can describe anatomical or biological features such as differences in neurovascular coupling in the cortical layers, while other terms may be needed to describe the spatial scale of blood flow regulation, or to take technical properties into account such as the differential sensitivity of various fMRI techniques to arteries, veins and parenchyma, or the effect of increasing field strength. Increasing the field strength alters blood T_2_ and T_2_^*^ and increases the size and spatial extent of susceptibility gradients. Furthermore, the angle of the vessel to the main magnetic field affects the BOLD signal, with the parallel orientation leading to a lack of signal attenuation (Ogawa et al., 1993).

The signal measured with BOLD fMRI originates from changes in the deoxyhemoglobin (dHb) concentration, and is therefore sensitive to changes in cerebral blood flow (CBF), blood volume (CBV), and tissue oxygen consumption. In healthy brain, neural activity typically leads to a concurrent increase in oxygen consumption, blood flow, and volume. The inflow of oxygenated blood exceeds the O_2_-consumption, leading to a reduction in the dHb-concentration and an increase in image intensity. Therefore, the BOLD response needs to be modeled as a function of both metabolism and blood flow (Buxton et al., 1998, 2004; Stephan et al., 2007; Vazquez et al., 2008). Furthermore, the changes in the local magnetic field induced by the paramagnetic dHb are different for intravascular and extravascular water, and the local magnetic field changes also depend on the size and orientation of the blood vessel, the distance to the blood vessel and the magnetic field strength (Ogawa et al., 1993). A description of BOLD models is beyond the scope of this review, but we describe some basic properties of compartmental models that are often used for modeling the hemodynamic response (Buxton et al., 2004; Zheng et al., 2005; Boas et al., 2008; Fantini, 2014; Heinzle et al., 2016; Markuerkiaga et al., 2016).

Different compartments can be used to model the different vascular elements (arteries, capillaries, veins), or vasculature vs. tissue, or different cortical layers. The general equation for the amount of deoxyhemoglobin (Q) in a compartment is a function of the dHb entering and leaving the compartment and its production:

$$\begin{array}{l} \frac{dQ}{dt}=F_{in}\left(t\right)-\;F_{out}\left(Q,t\right)+F_{prod}\left(Q,t\right) \end{array}$$

leading to n coupled differential equations for n compartments. Note that the production term only arises for compartments containing cortical parenchyma, and that F_*in*_ and F_*out*_ are functions describing the amount of dHb entering and leaving the compartment, and thus these are CBF-dependent terms (*CBF* · [*dHb*]).

Some properties make fMRI modeling challenging, and additional terms or functions are needed to describe the system:

1. Vasodilation in response to neural activity renders the volume of compartments time-dependent, leading to time-dependent volume terms in the equations.
2. The MRI signal does not vary linearly with Q. The degree of signal attenuation following an increase in dHb differs for extravascular or intravascular water protons, or for water near capillaries or near veins, and depends on the size and orientation of the vessel (Ogawa et al., 1993; Kennan et al., 1994; Weisskoff et al., 1994; Boxerman et al., 1995). Furthermore, different sequences can be more or less sensitive to water near capillaries or near veins (see below).

The additional terms to describe these properties and the larger number of compartments required for laminar models, leads to more degrees of freedom in the model. Additional equations in the model are based on the conservation of mass and volume, e.g., $\frac{dV}{dt}={CBF}_{in}-{CBF}_{out}$, and possibly the Grubb relation: *CBV* ∝ *CBF*^α^ with α = 0.38 (Grubb et al., 1974). The large number of terms and equations may require assumptions to simplify the model and reduce the degrees of freedom. It is also advantageous to combine BOLD with other MRI methods such as functional CBF and CBV (Yang et al., 2004) because these provide additional information and simplify equations, given that the production term is absent. CBF can be measured using arterial spin labeling methods, while CBV uses contrast agents (Mandeville et al., 1998) or vascular space occupancy (VASO; Lu et al., 2003).

The description above briefly and generally describes the principles of any BOLD model. In this paper we give an overview of why high-resolution fMRI necessitates the expansion of current BOLD-models to incorporate additional terms and compartments, and what these additional terms relate to. We focus on biological and MR-issues that emerge at the mesoscopic scale, but we first discuss how combining BOLD fMRI with methods such as CBV and CBF can aid the interpretation of fMRI data and neurovascular coupling.

## Combining BOLD with CBV and CBV methods to investigate neurovascular coupling

CBF and CBV usually co-vary and are related to the cerebral metabolic rate of oxygen consumption (CMRO_2_). However, in some cases, for instance in disease, development, or after pharmacological intervention, CBV, CBF, and CMRO_2_ do not increase or decrease simultaneously (Kozberg et al., 2013; Hillman, 2014). This is important since it has been shown that neurovascular coupling and capillary function are impaired in a number of diseases, for instance Alzheimer's disease or small vessel disease, while for other diseases it is not known if and how neurovascular coupling is affected (Fleisher et al., 2009; Troprés et al., 2015; Østergaard et al., 2016). In these cases one cannot make inferences about metabolism from the BOLD response alone, and combination of BOLD fMRI with CBF or CBV can improve the interpretation of the signal.

**Figure 1:**
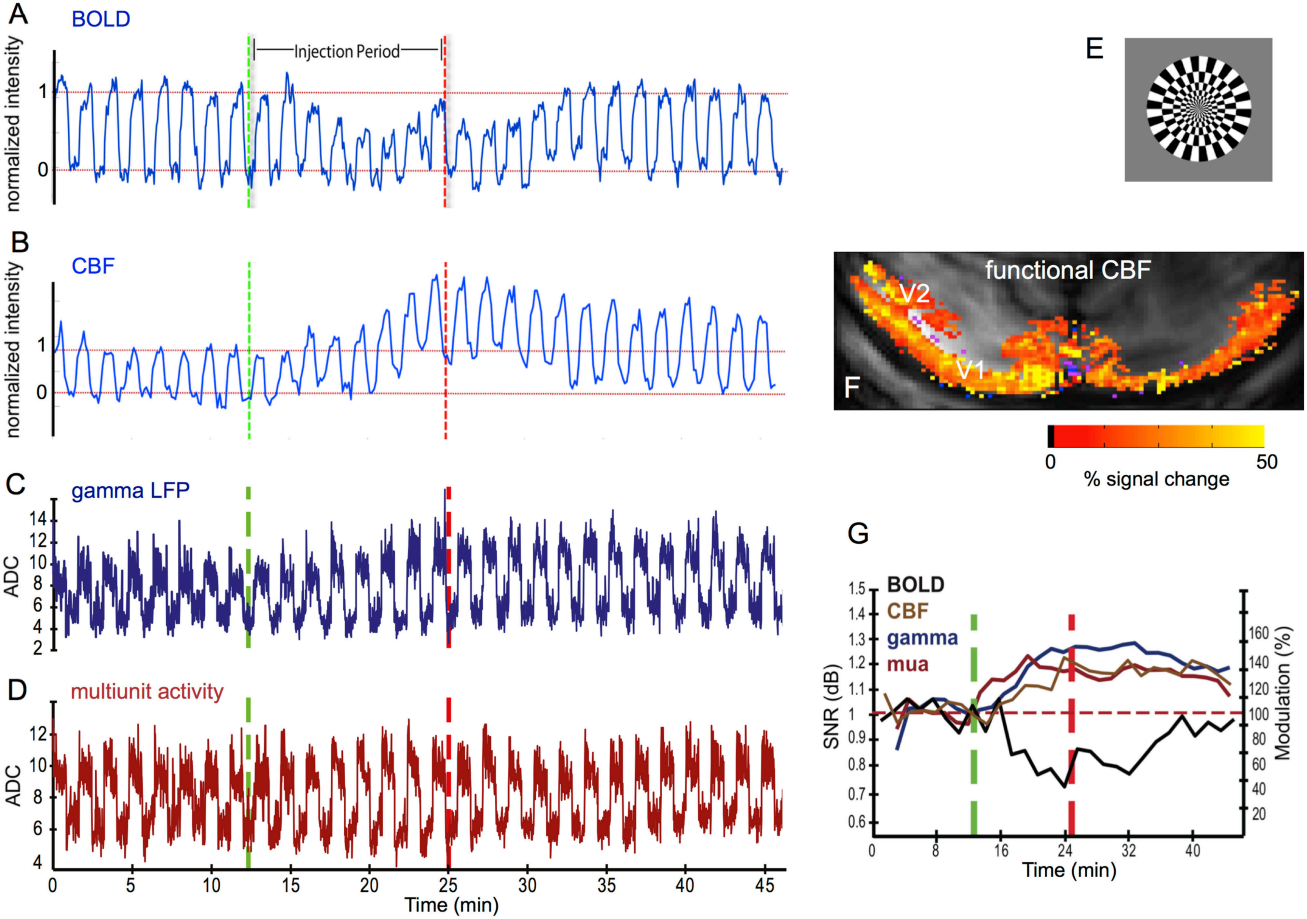
**Effect of systemic injection of dopamine on BOLD-, CBF-, and neural responses to a visual stimulus in V1 of anesthetized macaques**. Systemic dopamine injection reduced the BOLD response to a visual stimulus **(A)**, while the CBF **(B)**, gamma LFP (**C**, LFP 40–150 Hz), and multiunit activity **(D)** increased relative to the pre-injection level. fMRI and electrophysiological data were acquired in response to a full-field rotating black-and-white checkerboard stimulus **(E)**. **(F)** example CBF activation map, showing the functional CBF response in V1 an V2. **(G)** relative change in modulation of the neural- and fMRI responses after dopamine injection. Electrophysiology data were acquired using 16-contact NeuroNexus laminar electrodes and the traces **(C,D)** were averaged over all electrode sites. MRI-data were acquired at 7T, using GE-EPI for BOLD **(A,G)** and FAIR for CBF **(B,F,G)**. See (Zaldivar et al., 2014) for experimental details. Adapted from Zaldivar et al. (2014), with permission.

CBF and CMRO_2_ are under the influence of multiple regulatory pathways and are able to change independently. CBF and CBV can change without a metabolic change, such as under hypercapnia (Kety and Schmidt, 1948; Hafkenschiel and Friedland, 1952; Novack et al., 1953; Jones et al., 2001; Zappe et al., 2008c), and metabolism can change without a change in CBF, for example upon sensory stimulation when the vasculature is already maximally dilated (Nagaoka et al., 2006; Zappe et al., 2008b). This is most apparent when the BOLD response turns negative, but changes in coupling between flow and metabolism can also occur for positive BOLD. Figure 1 shows an example of the latter, where systemic injection of dopamine led to a reduction in the BOLD response in V1, while the neural- and CBF-responses were increased. A reduced BOLD response could be the result of a decrease in CBF with unchanged metabolism, or it could be due to an increase in metabolism (an increase in [dHb]), that is not offset by an equivalent increase in inflow of oxyhemoglobin. The increase in the neural response suggests an increase in metabolism that is not sufficiently matched by the increase in CBF, leading to an increase in [dHb] and a reduced BOLD response (Zaldivar et al., 2014).

**Figure 2:**
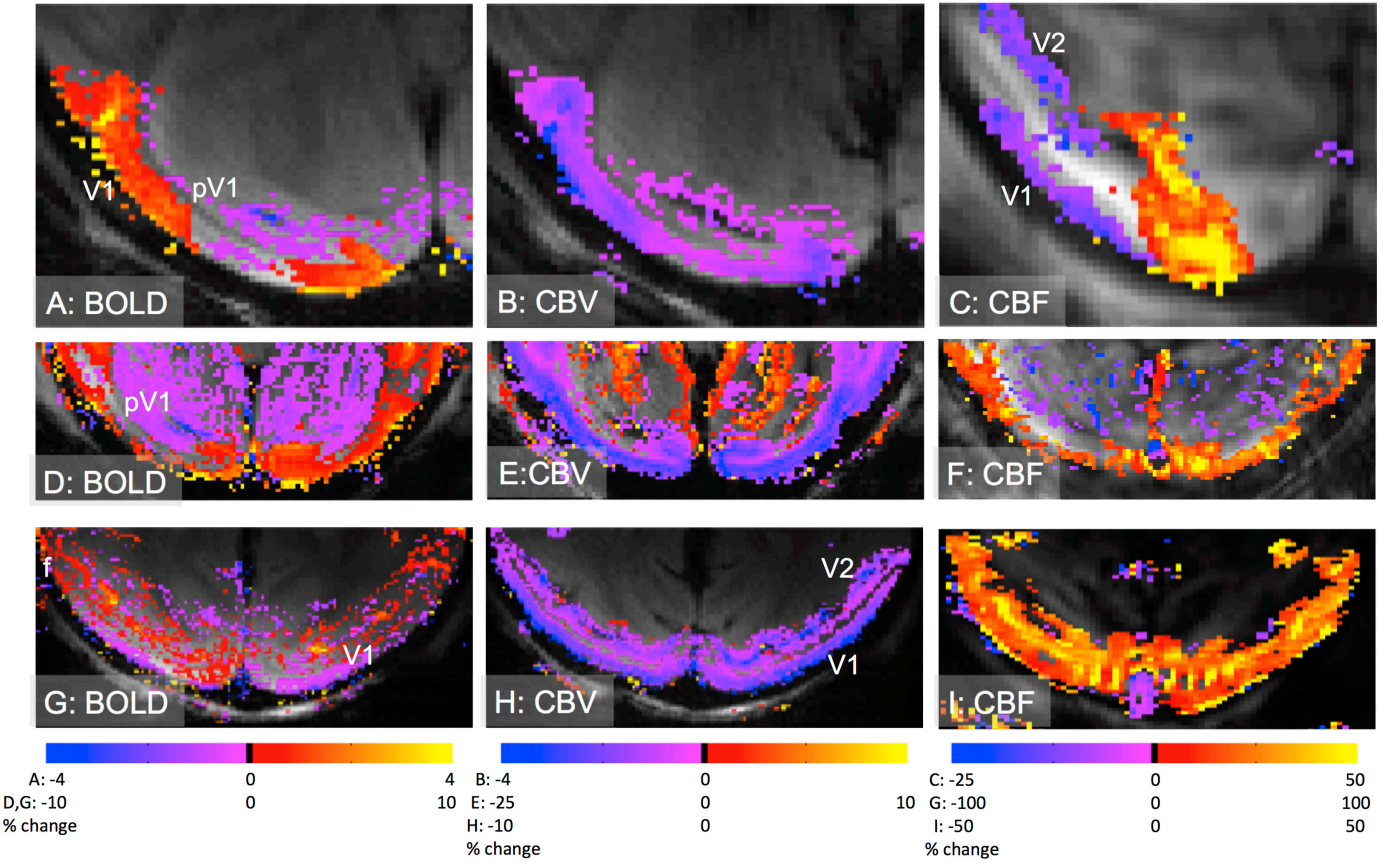
**Different types of negative BOLD responses in early visual cortex of anesthetized macaques**. **(A–C)**: negative BOLD adjacent to positive BOLD in V1 operculum **(A)** corresponds to an increase in CBV **(B)** and a decrease in CBF **(C)**. The contrast-agent based CBV-response is inverted and shows a signal decrease (violet) for an increase in CBV. The stimulus was a gapped rotating black-and-white checkerboard. Adapted from Goense et al. (2012), with permission. **(D–F)**: negative BOLD in peripheral V1 (“pV1”) and extrastriate areas such as visual area V2, in response to a full-field rotating black-and-white checkerboard. The negative BOLD signal **(D)** is associated with a decrease in CBV **(E)** and CBF **(F)**. Note that the negative CBF-signal does not show many significant voxels, despite having a sizable negative response, however this is the result of the high spatial resolution and the reduced sensitivity in the center of the brain due to its large distance from the receiver coils (Goense et al., 2010). **(G–I)**: negative BOLD response in V1 to a full-field rotating checkerboard stimulus **(G)**. The negative BOLD is associated with an increase in CBV **(H)** and CBF **(I)** as also seen in rodents (Schridde et al., 2008). Interestingly, the foveal area (“f”) showed a positive BOLD response. Acquisition parameters: **(A–C, G–I)** 4.7T, **(D–F)** 7T; for BOLD, 8-segment GE-EPI, resolution LRxAP 500 × 375 μm, TE 20 ms, TR 750 ms; for CBV, 8-segment GE-EPI, resolution 500 × 375 μm, TE 12–20 ms, TR 750 ms, 8 mg/kg MION **(B** and **H)** or Feraheme **(E)**; for CBF, FAIR-based ASL, single shot EPI, resolution 500 × 500 μm **(C)**, 500 × 312 μm **(F)** and 744 × 500 μm **(H)**, TE 9.5–12 ms, TI 925–1300 ms, TR 3000–4500 ms.

Differences in the mechanism for neurovascular coupling were also observed for the negative BOLD response. The mechanisms for the negative BOLD response seem more diverse than those of the positive BOLD response (Kim and Ogawa, 2012), although negative BOLD responses might just make the differences more obvious. Negative BOLD was first described as an initial dip preceding the positive BOLD, and a transient negative post-stimulus undershoot is also often observed response (Ernst and Hennig, 1994; Menon et al., 1995; Hu et al., 1997; Kim et al., 2000). Sustained negative BOLD responses were described somewhat later (Huang et al., 1996). Combining BOLD with other modalities allows differentiation between multiple mechanisms for negative BOLD, and the following mechanisms have been described:

- The initial dip, which is due to an initial stimulus-driven increase in CMRO_2_ while the CBF response lags the CMRO_2_ increase. This negative BOLD signal is transient and has also been observed with optical imaging (Chen-Bee et al., 2007).
- A negative BOLD response due to an increase in metabolism with an insufficient vascular response is related to the former mechanism. This can occur when CBF and CBV increases are insufficient to match the increase in CMRO_2_ (Figure 2) or in cases where there is a lack of CBF response (Nagaoka et al., 2006; Schridde et al., 2008).
- A negative BOLD response due to a reduction of neural activity that leads to a decrease in CBF accompanied by a decrease or increase in CBV (Figure 2; Shmuel et al., 2002, 2006; Devor et al., 2008; Boorman et al., 2010; Goense et al., 2012).
- A negative BOLD response due to a blood flow redistribution with a decrease in CBV that is also referred to as the “blood- steal” effect (Harel et al., 2002).
- Negative BOLD responses have been observed in subcortical areas concurrent with increases in neural activity and decreases in CBV (Shih et al., 2009).
- Negative BOLD responses have also been observed due to volume changes of the CSF (Bianciardi et al., 2011; Thomas et al., 2013).

Examples of different negative BOLD mechanisms in macaque visual cortex are shown in Figure 2. Rotating ring stimuli similar to those used in Shmuel et al. yield negative BOLD adjacent to positive BOLD (Figures 2A–C), associated with a decrease in neural activity and CBF (Shmuel et al., 2002, 2006), but unexpectedly an increase in CBV (Goense et al., 2012; Zaldivar et al., 2015). However, in the V1 periphery or in extrastriate areas, negative BOLD was associated with decreases in CBF and CBV (Figures 2D–F). In the latter case it is unknown whether neural activity is reduced in the locations showing negative BOLD. In the case of a “blood steal” no reductions in neural activity are expected. An increased CMRO_2_ with an insufficient blood supply led to a negative BOLD although CBF and CBV were increased (Figures 2G–I).

Uncoupling of CBV and CBF and laminar differences in neurovascular coupling have been observed (Goense et al., 2012). Figures 2A–C shows an increase in CBV in the middle cortical layers that was accompanied by a strong decrease in CBF occurring in the upper layers. This demonstrates the potential of high-resolution fMRI to separate different laminar and/or vascular compartments, and to determine laminar differences in neurovascular coupling, demonstrating the utility of high-resolution fMRI for improving our understanding of neurovascular coupling.

## Biological properties at the mesoscopic scale

Conventional fMRI methods typically show functional activation in relatively large activated areas and sometimes large veins. High-resolution fMRI is able to discriminate surface arteries and veins from cortical parenchyma, and can separate different layers and columns. The layers and columns of V1 show differences in metabolism and vascularization (Horton and Hubel, 1981; Zheng et al., 1991; Hevner and Wong-Riley, 1992; Fonta and Imbert, 2002; Weber et al., 2008). For instance, higher resting and stimulus induced metabolism in layer IV have been demonstrated with 2-deoxyglucose autoradiography and cytochrome oxidase reactivity in primate V1 (Horton and Hubel, 1981; Wong-Riley and Carroll, 1984; Tootell et al., 1988), while layer IV is also more densely vascularized to meet its higher metabolic needs (Duvernoy et al., 1981; Fonta and Imbert, 2002; Lauwers et al., 2008; Weber et al., 2008). Similarly the blobs in V1 have a slightly higher metabolism and vascularization than the surrounding parenchyma (Zheng et al., 1991; Keller et al., 2011).

The ability to resolve cortical layers and columns depends ultimately on the scale of the blood flow regulation; laminar and columnar fMRI can only visualize layers if the cortical vasculature is controlled at scales beyond the penetrating arterioles, i.e., at the level of precapillary arterioles and capillaries, and if the fMRI-method used is sensitive to that level. CBF is regulated at multiple levels ranging from the large arteries to the capillaries (Hamel, 2006). Dilation of arteries and arterioles increases blood flow, but the observation that individual barrels of the rodent barrel cortex could be distinguished with optical imaging despite that penetrating arterioles are spaced more widely apart than the size of the barrels indicates regulation at a finer level (Woolsey et al., 1996; Blinder et al., 2013). Evidence from intrinsic optical imaging and two-photon imaging shows that blood flow regulation in response to sensory stimulation occurs at the level of precapillary arterioles and capillaries (Mulligan and Macvicar, 2004; Vanzetta et al., 2005; Devor et al., 2007; Iadecola and Nedergaard, 2007; Attwell et al., 2010; Tian et al., 2010; Hillman, 2014). Astrocytic signaling and pericytes play important roles in mediating such highly localized vasodilatory responses (MacVicar and Newman, 2015). Pericytes were shown to regulate capillary diameter, and are damaged by ischaemia (Hall et al., 2014), indicating that these regulatory mechanisms may be selectively impaired in disease (Troprés et al., 2015; Østergaard et al., 2016).

Whether blood flow is regulated at the level of individual laminae is still unknown, although the fMRI evidence points in that direction (Goense et al., 2012; Yu et al., 2014). BOLD, CBV, and CBF responses are larger in the middle layers of primary sensory cortex compared to superficial and deep layers (Goense and Logothetis, 2006; Harel et al., 2006; Zhao et al., 2006; Zappe et al., 2008a; Goense et al., 2010; Yu et al., 2014); differences that can only arise if blood flow is regulated at a laminar level. Optical imaging is less suitable for showing laminar changes in blood flow, because light scattering in the cortex limits the depth down to which responses can be visualized. CBF responses in different layers may also be more interdependent than flow responses in different columns, given the anatomy of the cortical vasculature (Woolsey et al., 1996), but further work is needed to resolve the exact mechanisms and the interdependence of CBF in the different layers.

At the mesoscopic scale the properties of the vascular system also become heterogeneous with respect to the voxel size. The vascular anatomy, with large vessels on the cortical surface and the cortical parenchyma devoid of large vessels allows separation of large vessels and parenchyma at imaging resolutions in the range of hundreds of microns (Weber et al., 2008). The time courses of the BOLD and CBV-responses in the superficial and deeper layers were shown to differ, reflecting the different compositions of the superficial and deeper voxels (Yacoub et al., 2006). Since arterial blood enters the cortex from the surface to supply the deeper layers, and venules and veins drain back toward the surface, this makes the blood supply in the deeper layers dependent on the blood supply from the surface, and on the blood supply to other layers (Woolsey et al., 1996; Martín et al., 2013). Blood oxygenation is therefore also expected to vary with cortical depth. However, note that blood oxygenation and flow in the capillary bed are heterogeneous (Jespersen and Østergaard, 2012; Sakadžić et al., 2014).

The question how the BOLD signal relates to the underlying neural activity is not fully resolved for common voxel sizes (Logothetis, 2008), and remains at the laminar level. Furthermore, laminar differences may exist in the coupling between neural activity and the hemodynamic response, given the differences in cell types and functionality of the cortical layers. Despite this, the ability to measure fMRI and electrophysiological signals in the cortical layers is expected to provide more detailed information about what neural processes drive the BOLD signal. However, the neural activity in the layers within a column is highly correlated, and stimulation induces responses throughout a column (Mountcastle, 1997). It is not usually possible to selectively activate an individual layer, and orthogonal stimuli such as those used to resolve ocular dominance or orientation columns are not typically available to resolve layers. However, clear differences in neural responses in the cortical layers exist and have been extensively studied in primate V1, where neurons with different stimulus specificity are found in different layers, such as sensitivity to color or motion (Tootell et al., 1988; Nassi and Callaway, 2009). It is not known exactly how the cortical network as a whole changes following stimulation, and how these changes in neural responses affect the hemodynamic response. There are indications that the laminar differences in neurovascular coupling are driven by differences in neural activity (Goense et al., 2012; Yu et al., 2014; Zaldivar et al., 2015). This provides the exciting prospect that laminar differences in neural processing, for instance the distinction between input and output, or feedforward and feedback, can be studied *in vivo* in humans with fMRI, although more work is needed to resolve the mechanisms.

**Figure 3:**
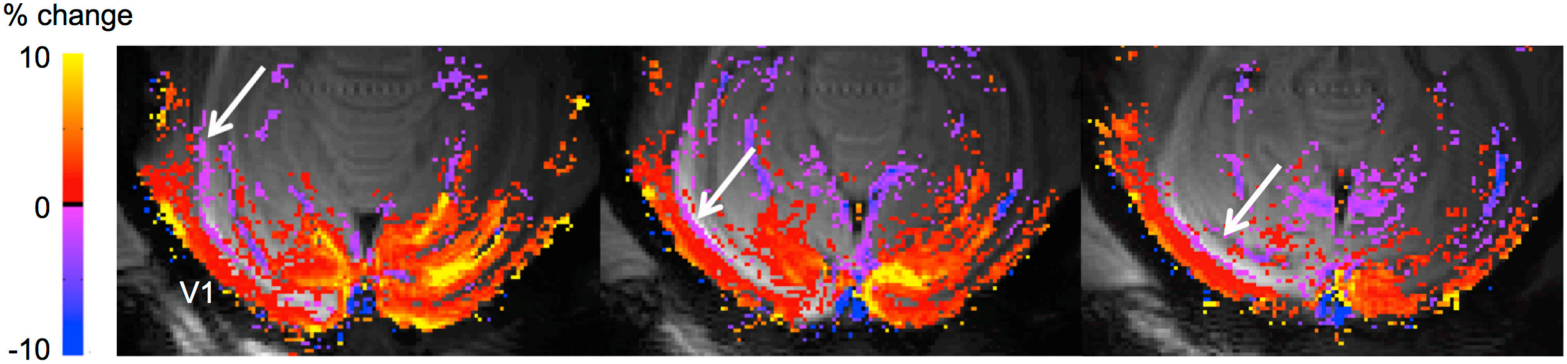
**Negative BOLD adjacent to the lateral ventricle in macaque V1**. The animal had an enlargement of the occipital horn of the left lateral ventricle, and negative BOLD can be seen at the border of the ventricle (white arrows) in the three slices covering the enlarged ventricle (note that normally the ventricle does not extend backward this far, as shown in the right hemisphere where the ventricle is not visible), as observed also in humans (van der Zwaag et al., 2009; Bianciardi et al., 2011). The stimulus was a full-field rotating checkerboard. Acquisition parameters: 4.7T, 8-segment gradient-echo EPI, resolution 500 × 500 μm, TE 20 ms, TR 750 ms.

A further factor that may come into play at the mesoscopic level is that increases in CBV due to functional activation can increase the total cortical volume (Jin and Kim, 2010; Bianciardi et al., 2011; Krieger et al., 2012; Thomas et al., 2013) leading to potentially spurious BOLD signals. Spurious negative BOLD responses have been observed near ventricles, and may be due to volume changes of the vascular and/or CSF compartments (Bianciardi et al., 2011; Thomas et al., 2013). Figure 3 shows an example of such a negative BOLD signal in macaque visual cortex, and it is apparent that it closely follows the borders of the ventricle.

To summarize, differences in biological properties at the mesoscopic scale can be visualized with high-resolution fMRI, and this may make it necessary to add compartments to models explaining neural and hemodynamic processes at high-resolution (Heinzle et al., 2016; Markuerkiaga et al., 2016).

## Laminar fMRI—technical challenges and specificity of fMRI methods

Several factors pertaining to resolution, sensitivity, and specificity of the acquisition methods also need to be addressed in the modeling of laminar fMRI responses. Laminar fMRI using BOLD, CBV and CBF has been shown in animals at resolutions of tens to hundreds of μm (Goense and Logothetis, 2006; Harel et al., 2006; Zhao et al., 2006; Zappe et al., 2008a; Goense et al., 2010; Yu et al., 2014), while laminar fMRI studies in humans are more recent (Koopmans et al., 2011; Huber et al., 2015). The achievable resolution in animal studies is higher than in humans due to the stronger gradients of animal scanners and the higher efficiency of the smaller, often custom-designed RF-coils (Logothetis et al., 2002; Doty et al., 2007; Goense et al., 2010). Laminar fMRI in humans has the advantage that it is done in awake subjects, although this leads to degradation of the resolution by motion. The weaker gradients on human scanners and the limitations of peripheral nerve stimulation (which limits the maximum gradient slew rate and maximum EPI gradient amplitude) restrict the maximum spatial resolution, and most laminar fMRI studies in humans use an isotropic resolution of 0.75–0.8 mm. For thinner cortical areas like primary sensory areas this may mean that few voxels span the cortex, and further increases in spatial resolution are desirable to better resolve the layers. The human brain is more gyrified than animal brains, necessitating isotropic resolution. This is compounded by the fact that the cortical thickness is not uniform, and tends to be thicker on the sulci compared to in the gyri, with most of the variability accounted for by variation in the thickness of the deeper layers, necessitating more sophisticated data-analysis procedures (Bok, 1929; Waehnert et al., 2014).

Laminar fMRI and its interpretation are also affected by the specificity of the acquisition method. The visibility of the BOLD-effect differs depending on the sequence and its parameters. For example, the high concentration of dHb or contrast agent in large vessels reduces the T_2_^*^ relaxation time to such an extent that little measurable signal remains. On the other hand, when vessels are parallel to the magnetic field, T_2_^*^ effects are reduced. Other examples are the lack of sensitivity of spin echo (SE)-BOLD to the static gradients around large vessels, or that at higher magnetic field strength the sensitivity of gradient echo (GE)-BOLD shifts from sensitivity to predominantly veins at low field to an increased sensitivity to capillaries at higher field (Kennan et al., 1994; Weisskoff et al., 1994; Boxerman et al., 1995). The specificity of the fMRI method to large vs. small vessels, or to extracellular vs. intracellular water therefore also needs to be taken into account in a model (Buxton et al., 1998; Uludag et al., 2009).

**Figure 4:**
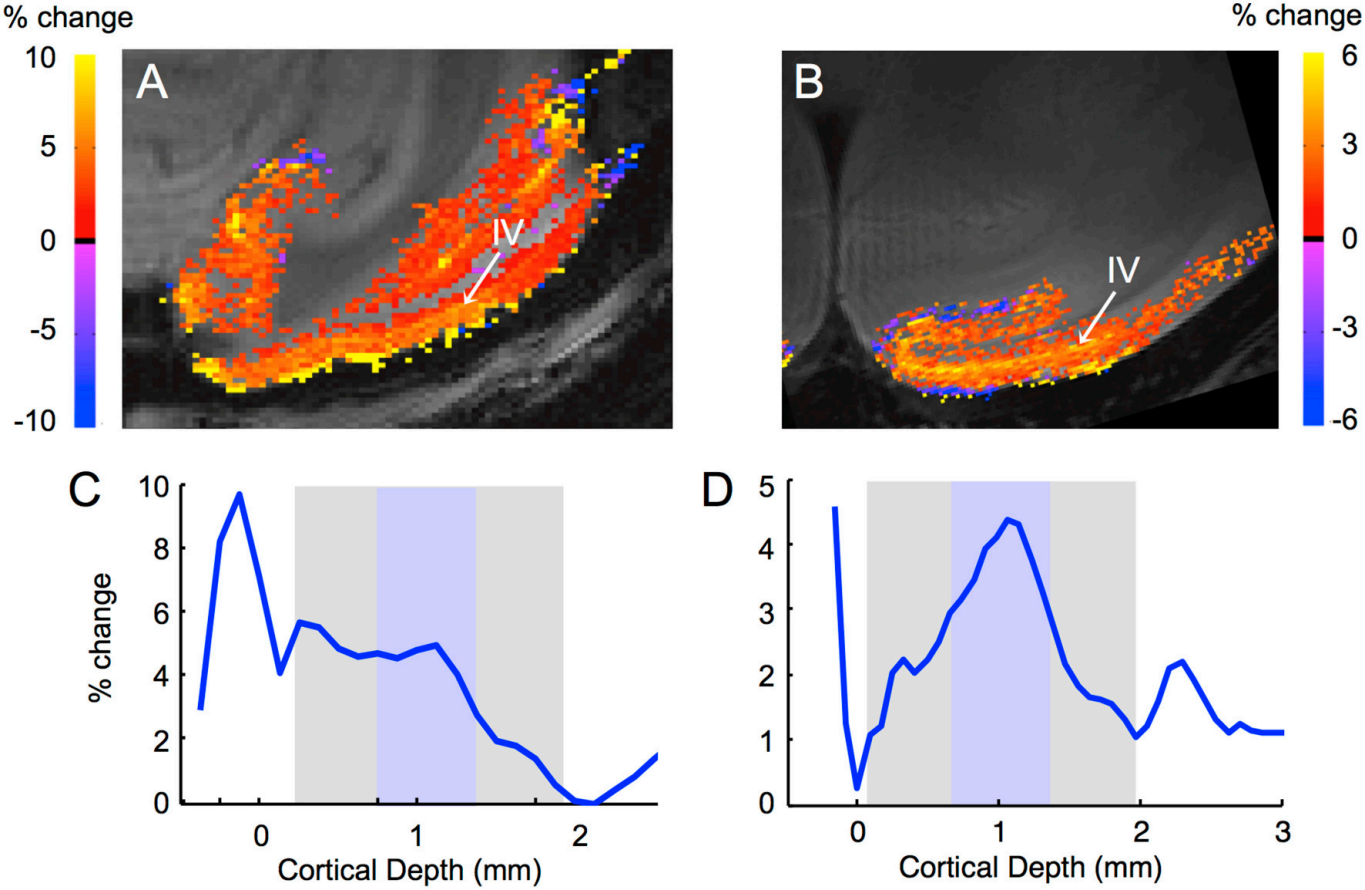
**Differences in laminar activation profiles for GE- and SE- BOLD fMRI**. High-resolution gradient-echo **(A)** and spin-echo **(B)** fMRI show different laminar activation profiles **(C,D)** in response to a full-field rotating black-and-white checkerboard stimulus. The GE-BOLD signal shows strong activation at the cortical surface, reflecting its high sensitivity to veins, while the SE-BOLD signal shows little sensitivity to the veins on the cortical surface. Both methods are sensitive to capillaries, evidenced by the higher activation of layer IV, the layer with the highest capillary density. Acquisition parameters: **(A)** 7T, 16-segment GE-EPI, resolution 333 × 250 μm, TE 20 ms, TR 750 ms. **(B)** 4.7T, 16-segment SE-EPI, resolution 250 × 187 μm, TE 46 ms, TR 2000 ms. **(C,D)** cortical profiles for GE-BOLD and SE-BOLD, respectively calculated perpendicular to the cortical surface (Goense et al., 2012). The cortical gray matter is indicated in gray with the approximate location of layer IV [based on the location of the Gennari line (Barbier et al., 2002; Logothetis et al., 2002)] in blue.

If an fMRI method is more sensitive to capillaries than to veins, the method is said to be more specific, since the location of the functional activation is presumably closer to the location of the neural activity. At higher resolution the sensitivity of different fMRI methods to different vascular compartments becomes visible, for instance, activation immediately adjacent to veins or arteries (Yu et al., 2012). In laminar fMRI this leads to different activation profiles for different fMRI methods; methods that are more sensitive to capillaries than to veins show maximal activation in the middle layers while methods that are more sensitive to veins show maximal activation at the cortical surface (Goense and Logothetis, 2006; Harel et al., 2006; Zhao et al., 2006; Zappe et al., 2008a; Goense et al., 2010, 2012; Yu et al., 2014; Huber et al., 2015). This is illustrated in Figure 4, which shows the different laminar profiles for GE-BOLD and SE-BOLD. GE-BOLD is sensitive to veins and capillaries, while SE-BOLD is most sensitive to capillaries (Kennan et al., 1994; Weisskoff et al., 1994; Boxerman et al., 1995). Figures 4A,C shows that the GE-BOLD response is strongest at the cortical surface where the pial veins are located, while the SE-BOLD is strongest in the middle of the cortex (Figures 4B,D). At 7T, layer IV can also be observed with GE-BOLD due to the larger capillary contribution at high field (Figure 4A), while at 4.7T layer IV is more difficult to distinguish (Goense et al., 2007). CBF and CBV methods also differ in specificity. CBF is sensitive to arterioles, capillaries and tissue water in exchange with water in these vessels. Contrast agent based CBV signal changes arise predominantly from water in or around smaller vessels (arterioles, venules, and capillaries) since signal from large vessels is lost due to the susceptibility effects arising from the high intravascular iron concentration. The same acquisition method can still show differences in specificity depending on sequence parameters; for instance, for BOLD-fMRI differences in laminar profiles have been shown due to differences in field strength, TE and segmentation (Goense and Logothetis, 2006; Koopmans et al., 2011), for CBF, due to labeling time (Zappe et al., 2008a), and for contrast agent based CBV methods, due to differences in field strength, TE or iron concentration.

The laminar sensitivity profiles of the fMRI signal do not usually affect regular fMRI (although sensitivity to veins can complicate signal localization when large sulcal veins are located near activated areas), but should be accounted for in laminar fMRI and high-resolution models. This can be done by including sensitivity to extravascular vs. intravascular water in the model, or by adding model parameters reflecting vessel size.

The previous section described sources of heterogeneity at the mesoscopic level. Biological sources of heterogeneity within the cortex and heterogeneity resulting from the properties of the acquisition methods were described. Although all these can in principle be parametrized in multicompartment models, this would lead to highly complex models with many degrees of freedom. Judicious choice of which additional model parameters to include, and which assumptions or simplifications are reasonable, based on the study questions and acquisition method is therefore paramount.

## Comparison with other methods and multimodal neurovascular coupling research

Many other techniques are used to study neurovascular coupling, such as optical imaging, functional photoacoustic imaging, functional ultrasound imaging, autoradiography, tracer techniques, histology, electrochemistry (e.g., probes to measure PO_2_), etc., and these techniques often provide complementary information. Optical imaging is used to answer questions about cortical function and the hemodynamic response, and the dynamics of BOLD and optical imaging responses are often compared. There are similarities but also differences between the methods and in the ideal case, one can inform the other.

The optical field has contributed much to the elucidation of the BOLD mechanism. Intrinsic optical imaging, in which tissue is illuminated and reflected light is measured, has superior in-plane resolution and the ability to discriminate oxygenated and deoxygenated hemoglobin. Arteries and veins are easily resolved, while the more diffuse background signal originates from capillaries. These properties, and because combination with CBF measurement and electrophysiology is straightforward, make it a valuable tool for comparison with fMRI, and the availability of both the oxy- and deoxyhemoglobin signal aids in making inferences about metabolism. The drawback of intrinsic optical imaging is that it is dominated by signal from the cortical surface and superficial layers. Although some signal originates from deeper layers, resolving power in the depth-dimension is absent (Hillman, 2007).

Two-photon imaging can reach cellular resolution and has greatly increased our understanding of neurovascular coupling. Single microvessels can be imaged *in vivo*, pinpointing the exact location of vasoconstriction and dilation and the signaling pathways involved, as well as flow in microvessels (Kleinfeld et al., 1998; Mulligan and Macvicar, 2004; Tian et al., 2010; Lecoq et al., 2011; Shih et al., 2012; Hillman, 2014). Its greatest drawback is light scattering in the tissue which limits the penetration depth to typically ~600 μm, corresponding to layer II/III (Hillman, 2007), while cutting-edge multi-photon methods are able to image down to ~1.2 mm. The latter covers the entire cortical thickness in the mouse (Mittmann et al., 2011; Hamel et al., 2015) although the cortex of rats and primates is substantially thicker. Optical coherence tomography (OCT) is a promising technique that is able to reach a depth of up to 2 mm, although its spatial resolution is diminished compared to other optical methods (Huang et al., 1991; Hillman, 2007; Srinivasan et al., 2009).

Neurovascular coupling research benefits from the comparison of optical imaging and high-resolution fMRI. However, optical methods' lack of resolving power in the depth dimension also complicates the comparison. fMRI voxels typically cover the entire cortical thickness, but their signal is compared with hemodynamic responses measured with optical imaging derived from the cortical surface and superficial layers. This comparison is only valid when the responses of the deeper layers are the same as those of the surface layers. Laminar fMRI can provide additional information by virtue of its ability to separate surface- and deep voxels and can thereby improve the comparison with optical imaging. The ability to resolve single arteries and venules with fMRI (Yu et al., 2012) can also make comparisons with optical imaging more accurate, as will improvements in the depth resolution of optical imaging (e.g., OCT).

The interpretation of neurovascular coupling at the mesoscopic scale is also expected to improve by comparison with new methods such as functional ultrasound and functional photoacoustic imaging that can measure blood flow, volume and oxygenation at high spatial and temporal resolution (Macé et al., 2011; Errico et al., 2015; Sieu et al., 2015; Yao et al., 2015; Demené et al., 2016) and by the increasing use of highly parallel electrophysiological recording methods, such as depth array electrodes, dense planar electrode arrays or dense ECoG grids in animals and humans (Viventi et al., 2011; Berényi et al., 2014; Chang, 2015; Kiani et al., 2015). These methods provide opportunities to compare neural and hemodynamic activity by increasing the electrophysiological sampling density and by more accurate electrode localization. Aside from its superior temporal resolution, the local field potentials (LFP) recorded with electrophysiology can be decomposed in different frequency bands that are engaged in different cognitive processes (Belitski et al., 2008). Low-frequency LFP information is more spatially diffuse than spiking and high frequency LFP information (Goense and Logothetis, 2008; Lindén et al., 2011; Einevoll et al., 2013), although the spatial extent of these signals is still relatively unknown. The neural responses recorded with multisite electrodes (depth-dependent or area-dependent) can be compared with high-resolution fMRI to provide information about laminar circuits (Maier et al., 2011; Zaldivar et al., 2015; Ma et al., 2016). The ability to record different locations simultaneously, such as different layers, or areas with positive and negative BOLD, would improve our understanding of neurovascular coupling. As fMRI increases its resolution and electrophysiology increases its coverage, this promises major advances in determining cortical circuit function *in vivo.*

Further exciting developments are the imaging and modeling of entire vascular and neural networks, which allows building increasingly sophisticated models of the cortical anatomy and blood flow in the cortical network (Guibert et al., 2010; Kleinfeld et al., 2011; Lorthois et al., 2011; Blinder et al., 2013; Kasthuri et al., 2015). Such multidisciplinary research and collaborations will allow us to study neurovascular coupling at increasingly fine spatial detail and build comprehensive models of neural circuit activity and the subsequent hemodynamic responses.

## Summary

In this paper we discussed the properties of high-resolution fMRI and factors that need to be taken into account when extending current macroscopic fMRI models to the mesoscopic scale that becomes accessible with high-resolution fMRI. Those factors are biological, for instance laminar differences in neurovascular coupling that become visible at high-resolution, and methodological, like the differences between GE- and SE-BOLD acquisitions. For modeling of BOLD-responses at the mesoscopic scale this means that additional compartments and variables need to be included, needing more elaborate models and judicious choice of the models' assumptions. Aside from this complication, high-resolution fMRI provides an opportunity to improve BOLD-models, since more information is available. An obvious example is the information about different cortical layers that becomes available, but high-resolution fMRI can also separate the responses in vessels from the responses in parenchyma and reduce partial volume effects. The extension of fMRI models to the mesoscopic scale holds the promise of better understanding of the BOLD signal, neurovascular coupling, and the possibility of elucidating neural circuitry *in vivo*.

## Author contributions

Acquired data: JG, YB, provided resources and supervision: NL, wrote first draft: JG. All authors edited and approved the manuscript.

### Conflict of interest statement

The authors declare that the research was conducted in the absence of any commercial or financial relationships that could be construed as a potential conflict of interest.
